# Roll Angular Rate Measurement for High Spinning Projectiles Based on Redundant Gyroscope System

**DOI:** 10.3390/mi11100940

**Published:** 2020-10-16

**Authors:** Jing Mi, Jie Li, Xi Zhang, Kaiqiang Feng, Chenjun Hu, Xiaokai Wei, Xiaoqiao Yuan

**Affiliations:** 1 National Key Laboratory for Electronic Measurement Technology, North University of China, Taiyuan 030051, China; s1806038@st.nuc.edu.cn (J.M.); b1506011@st.nuc.edu.cn (K.F.); b1806023@st.nuc.edu.cn (X.W.); s1906172@st.nuc.edu.cn (X.Y.); 2 School of Electrical Control Engineering, North University of China, Taiyuan 030051, China; zhangxi@nuc.edu.cn; 3 Suzhou Fashion Nano Technology Co., Ltd., Suzhou 215000, China; hcj74839@gmail.com

**Keywords:** precision-guided, roll angular rate, optimization-based angular rate estimation (OBARS), autoregressive integrated moving average (ARIMA), Sage-Husa Adaptive Kalman Filter (SHAKF)

## Abstract

Precision-guided projectiles, which can significantly improve the accuracy and efficiency of fire strikes, are on the rise in current military engagements. The accurate measurement of roll angular rate is critical to guide a gun-launched projectile. However, Micro-Electro-Mechanical System (MEMS) gyroscope with low cost and large range cannot meet the requirement of high precision roll angular rate measurement due to the limitation by the current technology level. Aiming at the problem, the optimization-based angular rate estimation (OBARS) method specific for projectiles is proposed in this study. First, the output angular rate model of redundant gyroscope system based on the autoregressive integrated moving average (ARIMA) model is established, and then the conventional random error model is improved with the ARIMA model. After that, a Sage-Husa Adaptive Kalman Filter (SHAKF) algorithm that can suppress the time-varying process and measurement noise under the flight condition of the high dynamic of the projectile is designed for the fusion of dynamic data. Finally, simulations and experiments have been carried out to validate the performance of the method. The results demonstrate the proposed method can effectively improve the angular rate accuracy more than the related traditional methods for high spinning projectiles.

## 1. Introduction

As a means of improving the accuracy and efficiency of firepower strike, precision-guided munitions has attracted the attention of the Army [1,2,3]. Extremely high spin rates complicate the guidance problem for precision-guided munitions [4]. The rotation rate of most projectiles can reach 10−30 rounds per second (r/s) for flight stability, and the launch overload can reach more than $10^{4}m/s^{2}$. Due to these characteristics of the projectile working environment, the attitude measurement method should meet the requirements of high accuracy, anti-overload, high output rate, and small in occupation. Therefore, the accurate measurement of projectile attitude as the basis of precise guidance and control has been regarded as a difficult task for a long time [5]. 

In order to measure the attitude of the high-spin projectile, various approaches have been proposed by numerous scholars. According to published literatures, the main sensors used for high-spin attitude measurement are as follow: solar azimuth sensor [6], continuous-wave radar [7], infrared sensor [8], Global Navigation Satellite System (GNSS) [9], magnetometer [10], accelerometer [11], gyroscope, [12] and so on. The measurement based on the solar azimuth sensor has high requirements for the visible conditions of the atmosphere. It may not work well when the sun is not visible. Infrared sensors can only be used in the see-sky background and are easily disturbed by other objects, so the application scenarios are greatly limited. GNSS attitude measurement system, compared with the inertial navigation system (INS), has the advantages of high precision, high reliability, and no time accumulated error. To meet the requirements of the attitude measurement of the projectile, three satellite receivers are used at least. However, the large volume of the satellite receiver is not suitable for the limited space of the projectile. The magnetometer based on the Earth’s magnetic field has the advantages of low cost, strong overload resistance, and all-weather operation [13,14,15]. On the one hand, the calibration of the magnetometer is complicated. On the other hand, the magnetometer is easily disturbed by the external environment and eddy current. Accelerometers can obtain the attitude by measuring the gravity component in the projectile coordinate. However, due to the complex motion of the projectile in flight, it is impossible to determine its attitude by gravity vector. Gyroscopes fabricated by microelectromechanical system (MEMS) technology are attractive due to their low cost, small size, and high output rate [16]. In attitude measurement, gyroscopes can be characterized as (a) autonomous measurement, (b) high reliability, and (c) simple algorithm. Due to these advantages, the MEMS gyroscope is applicable in various environments [17,18]. However, when the projectile flies at high speed, the bias of the selected large-range gyroscopes will be accumulated rapidly over time and leads to large attitude errors [19]. Thus, it is a challenging task for MEMS gyroscopes with the traditional configuration scheme to measure the angular rate of the high-spin projectile accurately.

To solve the problem that the large-range gyroscope cannot measure the angular rate of high spinning projectiles, the redundant sensor approach is generally used to enhance their accuracy [20,21,22,23]. The core of this method is the establishment of model and the fusion of redundant sensor output data [24]. However, the existing redundant sensor methods which cannot be applied in the high spinning and the high dynamic of projectiles have two defects: (1) the configuration method of small-range gyroscopes is not suitable for the high spinning state of projectiles. In addition, the common random error model of MEMS gyroscopes only considers the rate random walk (RRW) and angle random walk (ARW) ignoring other noise items. Furthermore, MEMS gyroscope’s output signal has a weak linear trend item [25,26,27]; (2) the existing fusion method uses the Conventional Kalman Filter (CKF) algorithm [28,29,30]. The premise of CKF to obtain the optimal estimation is that the structural parameters and statistical noise parameters of stochastic dynamic systems need to be known accurately [31]. However, in the flight condition of the high dynamic of the projectile, fixed noise parameters may cause unreliable results, which could lead to filter divergence.

In this paper, the optimization-based angular rate estimation (OBARS) method is proposed with an application to the high-spin projectiles. The main contributions of our research are twofold—(1) the gyroscopes geometry configuration of the inertial measurement unit (IMU) is designed according to the high dynamic characteristics of projectiles. In this configuration method, the output angular rate model of redundant gyroscope system based on the autoregressive integrated moving average (ARIMA) model is established, and then the conventional random error model is improved with the ARIMA model; (2) a Sage-Husa Adaptive Kalman Filter (SHAKF) algorithm that can suppress the time-varying process and measurement noise under the flight condition of the high dynamic of the projectile is designed for the fusion of dynamic data. By estimating the error of the gyroscope, compensating, and correcting the measurement information, the high-precision estimation of the angular rate is obtained. The OBARS method is verified by simulations and experiments in both static and dynamic states. The results show that the proposed method is superior to the traditional method.

This paper is organized as follows. In Section 2, based on the establishment of conventional gyroscope random error model, the calculation of roll angular rate is briefly introduced. In Section 3, according to the high dynamic state of projectiles, the improved gyroscope random error model and a Sage-Husa Adaptive Kalman Filter (SHAKF) algorithm is introduced in detail. The results of simulations and experiments are used to verify the performance of the method and are shown in Section 4. Finally, the article is concluded in Section 5.

## 2. Effect of Gyroscope Random Error on Angular Rate Accuracy

### 2.1. Conventional Random Error Model of Gyroscope

**Figure 1:**
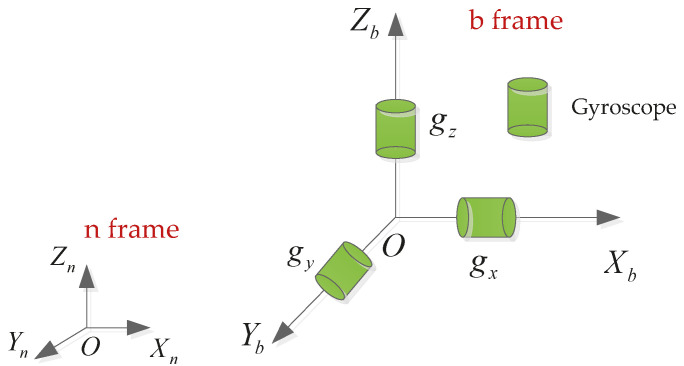
General sensor configuration.

The traditional measurement method of projectile angular rate is to install an angular rate sensor on each axis. Three single-axis gyroscopes mounted on the three-axes of the projectile, respectively, along with the different reference frames used in the analysis is shown in Figure 1. The local geographical coordinate is selected as the navigation frame denoted by n with origin $O_{n}$ and axes denoted by $X_{n}$, $Y_{n}$, and $Z_{n}$. The $X_{n}$-axis, $Y_{n}$-axis and $Z_{n}$-axis point to the east, north, and up, respectively. The inertial measurement unit (IMU) frame fixed on the carrier is selected to the b frame with a set of axes ($X_{b}$-$Y_{b}$-$Z_{b}$). The $X_{b}$-axis points to the right, $Y_{b}$-axis points to the front, and $Z_{b}$-axis points to the north. In this article, the direction cosine matrix (after this referred to as the attitude matrix) is used for representing the transformation between two frames which are respectively denoted by the superscripts and the subscripts of the attitude matrix. 

The angular rate of the projectile measured by gyroscopes is defined as

(1) $$\omega_{ib}^{b}=\omega_{in}^{b}+\omega_{nb}^{b},$$

(2) $$\omega_{in}^{b}=\omega_{ie}^{b}+\omega_{en}^{b},$$

where $\omega_{ie}$ is the rotation angular velocity of the earth, $\omega_{en}$ is the angular rate produced by the rotation of the navigation coordinate system on the earth due to the motion of the carrier, $\omega_{nb}$ is the attitude rate.

As an angular motion measurement device of carrier, a gyroscope has a direct impact on the attitude error of high spinning projectile system. Gyroscope measurements are usually corrupted with sensor noise. It is advisable to reduce the noise level of the MEMS gyroscope signals before using the measurements. Furthermore, a suitable modeling of these errors is vital to guarantee the system performance. Most of the standard gyroscope de-noising methods cannot effectively reduce the random noise level of MEMS gyroscope measurement without destroying the useful information in the sensor signal. According to the literature [32], the stochastic error of gyroscope is modeled as the Equations (3)–(5).

(3) $${\dot{\varepsilon }}_{b}=0,$$

(4) $${\dot{\varepsilon }}_{r}=-\frac{1}{\tau }\cdot \varepsilon_{r}+w_{r},$$

(5) $$\varepsilon_{c}=\varepsilon_{b}+\varepsilon_{r}+w_{g},$$

$\varepsilon_{b}$ is the successive starting drift. $\varepsilon_{r}$ described by a first order Markov process is a slowly varying drift as shown in Equation (4) above, where τ is the correlation time and $w_{r}$ is the driving white noise. $\varepsilon_{c}$ is the total random drift error of the gyroscope, and $w_{g}$ is a white noise process.

When the correlation time is long, that is, when the gyroscope working time is far less than the correlation time, the correlation drift can be approximately regarded as a random constant and incorporated into the successive starting error of the gyroscope, as shown in Equation (6).

(6) $$\varepsilon_{b}^{\prime }=\varepsilon_{b}+\varepsilon_{r},$$

If the first order Markov process is approximated to random walk ξ, then $\varepsilon_{r}$ can be expressed as

(7) $${\dot{\varepsilon }}_{r}=\xi ,$$

Therefore, the error model of gyroscope is shown in Equation (8).

(8) $$\varepsilon_{c}=\varepsilon_{b}^{\prime }+w_{g},$$

Consistent with the gyroscope error model shown in Equation (5), the gyroscope output is shown in Equation (9) [33].

(9) $$\left\{ \begin{array}{l} y=b+\omega +n_{a}\\ \dot{b}=n_{r} \end{array}\right.,$$

where b is the drift-rate bias, $n_{a}$ is the drift-rate noise. $n_{a}$ is assumed to be a Gaussian white noise process.

(10) $$E\left[n_{a}\left(t\right)\right]=0,E\left[n_{a}\left(t\right)n^{T}{}_{a}\left(t+\tau \right)\right]=R_{k}\delta \left(\tau \right),$$

δ(τ) is Kronecker delta function.

(11) $$\delta \left(\tau \right)=\{\begin{array}{cc} 1 & \tau =0\\ 0 & otherwise \end{array},$$

(12) $$E\left[n_{r}\left(t\right)\right]=0,E\left[n_{r}\left(t\right)n^{T}{}_{r}\left(t+\tau \right)\right]=Q_{k}\delta \left(\tau \right),$$

The two noise processes are assumed to be uncorrelated.

(13) $$E\left[n_{a}\left(t\right)n^{T}{}_{r}\left(t+\tau \right)\right]=0,$$

### 2.2. Problem Formulation

The Euler sequence of rotations (Z−X−Y) is employed to define the orientation of the projectile with respect to the n frame in terms of roll (γ), pitch (θ), and yaw (ψ) angles. The attitude matrix from n frame to b frame can be written as

(14) $$C_{n}^{b}=C_{2}^{b}C_{1}^{2}C_{n}^{1},$$

where

(15) $$C_{n}^{1}=\left[\begin{array}{ccc} \cos\psi & -\sin\psi & 0\\ \sin\psi & \cos\psi & 0\\ 0 & 0 & 1 \end{array}\right],\;C_{1}^{2}=\left[\begin{array}{ccc} 1 & 0 & 0\\ 0 & \cos\theta & \sin\theta \\ 0 & -\sin\theta & \cos\theta \end{array}\right],\;C_{2}^{b}=\left[\begin{array}{ccc} \cos\gamma & 0 & -\sin\gamma \\ 0 & 1 & 0\\ \sin\gamma & 0 & \cos\gamma \end{array}\right],$$

The component of attitude rate $\omega_{nb}$ (b frame relative to n frame) in the b frame is given by

(16) $$\begin{array}{c} \left[\begin{array}{c} \omega_{nbx}^{b}\\ \omega_{nby}^{b}\\ \omega_{nbz}^{b} \end{array}\right]=C_{1}^{b}\left[\begin{array}{c} \dot{\theta }\\ 0\\ -\dot{\psi } \end{array}\right]+\left[\begin{array}{c} 0\\ \dot{\gamma }\\ 0 \end{array}\right]=\left[\begin{array}{ccc} \cos\gamma & \sin\theta \sin\gamma & -\cos\theta \sin\gamma \\ 0 & \cos\theta & \sin\theta \\ \sin\gamma & -\sin\theta \cos\gamma & \cos\theta \cos\gamma \end{array}\right]\left[\begin{array}{c} \dot{\theta }\\ 0\\ -\dot{\psi } \end{array}\right]+\left[\begin{array}{c} 0\\ \dot{\gamma }\\ 0 \end{array}\right]\\ =\left[\begin{array}{c} \dot{\theta }\cos\gamma +\dot{\psi }\cos\theta \sin\gamma \\ -\dot{\psi }\sin\theta +\dot{\gamma }\\ \dot{\theta }\sin\gamma -\dot{\psi }\cos\gamma \cos\theta \end{array}\right]=\left[\begin{array}{ccc} \cos\theta \sin\gamma & \cos\gamma & 0\\ -\sin\theta & 0 & 1\\ -\cos\theta \cos\gamma & \sin\gamma & 0 \end{array}\right]\left[\begin{array}{c} \dot{\psi }\\ \dot{\theta }\\ \dot{\gamma } \end{array}\right] \end{array}$$

Therefore,

(17) $$\left[\begin{array}{c} \dot{\psi }\\ \dot{\theta }\\ \dot{\gamma } \end{array}\right]={\left[\begin{array}{ccc} \cos\theta \sin\gamma & \cos\gamma & 0\\ -\sin\theta & 0 & 1\\ -\cos\theta \cos\gamma & \sin\gamma & 0 \end{array}\right]}^{-1}\left[\begin{array}{c} \omega_{nbx}^{b}\\ \omega_{nby}^{b}\\ \omega_{nbz}^{b} \end{array}\right]=\left[\begin{array}{ccc} \frac{\sin\gamma }{\cos\theta } & 0 & -\frac{\cos\gamma }{\cos\theta }\\ -\cos\gamma & 0 & \sin\gamma \\ \sin\gamma \tan\theta & 1 & -\cos\gamma \tan\theta \end{array}\right]\left[\begin{array}{c} \omega_{nbx}^{b}\\ \omega_{nby}^{b}\\ \omega_{nbz}^{b} \end{array}\right],$$

Equation (17) can be converted into the following.

(18) $$\dot{\psi }=\frac{\sin\gamma }{\cos\theta }\omega_{nbx}^{b}-\frac{\cos\gamma }{\cos\theta }\omega_{nbz}^{b},$$

(19) $$\dot{\theta }=\cos\gamma \omega_{nbx}^{b}+\sin\gamma \omega_{nbz}^{b},$$

(20) $$\dot{\gamma }=\sin\gamma \tan\theta \omega_{nbx}^{b}+\omega_{nby}^{b}-\cos\gamma \tan\theta \omega_{nbz}^{b},$$

According to Equations (18)–(20), the attitude angle of the carrier is directly calculated by solving the Euler angle differential equation with angular rate $\omega_{nb}$. Therefore, it is vital to obtain the attitude rate $\omega_{nb}$. The projectile rotates at high speed during flight. Gyroscopes as a typical angular rate sensor need a large range for the high rotation speed of the projectiles. For the traditional angular rate measurement method, the use of large range gyroscope cannot meet the requirements of accurate attitude measurement. In addition, MEMS gyroscopes are characterized by high noise and large uncertainties, such as random walk, bias instability, and quantization noise in their outputs. All these factors are given a detailed annotation in IEEE Standards [34]. In practice, these errors will increase rapidly in a short time and reduce the precision of gyroscope. In the attitude solution for carrier, gyroscope noise is integrated into the attitude algorithm and these errors are accumulated, leading to a significant drift in velocity and position output. Therefore, a more efficient method should be proposed to offset the shortage. 

## 3. The Proposed Roll Angular Rate Measurement Method

In this section, the optimization-based angular rate estimation (OBARS) method for the high-spin projectile is proposed. This method falls into two portions—(1) improved random error model for high spinning projectiles based on the ARIMA model is established; (2) a Sage-Husa Adaptive Kalman Filter (SHAKF) algorithm that can suppress the time-varying process and measurement noise under the flight condition of the high dynamic of the projectile is designed for the fusion of dynamic data. 

### 3.1. ARIMA Model

In an ARIMA(p,d,q) model [35], the observation at time k is assumed to be a linear function of several past observations and random errors. Furthermore, time series can be predicted by using the ARIMA model. Therefore, the autoregressive integrated moving average (ARIMA) model suitable for the high dynamic of high-spin projectiles is used to model the time series $\left\{x_{k}\right\}$ of the redundant gyroscope system output as shown in Equation (21).

(21) $$\Phi \left(B\right)\nabla^{d}x_{k}=\Theta \left(B\right)a_{k},$$

(22) $$\begin{array}{l} \Phi \left(B\right)=1-\varphi_{1}B-\cdots -\varphi_{p}B^{p}\\ \Theta \left(B\right)=1-\theta_{1}B-\cdots -\theta_{q}B^{q} \end{array},$$

where $\nabla^{d}={\left(1-B\right)}^{d}$, Φ(B) is an the autoregressive coefficient, and Θ(B) is a moving smoothness coefficient. Operator

B

represents the backward shift operator, p is referred to as the order of the autoregressive model, q is referred to as the order of the moving average model, and d is referred to as order of differencing. White noise, $a_{k}$, are assumed to be independently and identically distributed with mean zero and constant variance $\sigma_{a}^{2}$. 

In the flight of projectile, the gyroscope output data is non-stationary. Furthermore, stationary data is obtained by first-order difference. The higher the order of ARIMA, that is, the greater the value of p and q, the better the characteristics of redundant gyroscope system output data can be described. However, with the increase in the order, the dimension of filtering calculation will greatly increase. Through the comprehensive analysis of five groups experimental data, the ARIMA(2,1,1) model is chosen to model the time series $\left\{x_{k}\right\}$. Parameters in ARIMA(2,1,1) are as follows: $\varphi_{1}=0.2683,\varphi_{2}=-0.2416,\theta_{1}=-0.6754$.

(23) $$\begin{array}{cc} x_{k} & =\left(1+\varphi_{1}\right)x_{k-1}+\left(\varphi_{2}-\varphi_{1}\right)x_{k-2}-\varphi_{2}x_{k-3}+a_{k}-\theta_{1}a_{k-1}\\ & =1.2683x_{k-1}-0.5099x_{k-2}+0.2416x_{k-3}+a_{k}+0.6754a_{k-1} \end{array},$$

### 3.2. Improved Gyroscope Random Error Model for High Spinning Projectiles

**Figure 2:**
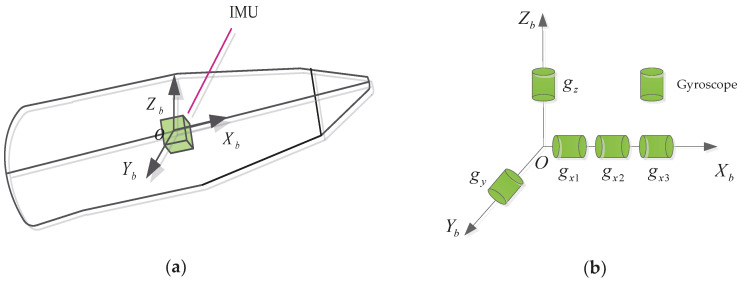
(**a**) The geometry of a projectile; (**b**) Geometry configuration.

Figure 2 shows the IMU and the geometry of a projectile. The IMU is fixed at the projectile mass center, with origin at the projectile center of gravity. $X_{b}$ is selected to be the spin axis of the projectile through point O. In IMU, the sensor configuration of $Y_{b}$-axis and $Z_{b}$-axis is the same as that of traditional scheme, that is, one gyroscope sensor is placed in each. Although the roll angular rate can be measured with a single gyroscope, multi gyroscope applications are better suited for projectiles that are undergoing a high-speed spinning flight. The geometric layout can make the most of the measurements provided by multiple sensors, so as to improve the accuracy of angular rate. Hence, three single-gyroscopes are placed parallel to the $X_{b}$-axis to improve the measurement accuracy of the roll axis. The gyroscopes geometry configuration is shown in Figure 2b.

The optimized roll angular rate is obtained by fusing the output data of three gyroscopes. The theoretical basis of improving angular rate precision is based on the correlation between gyroscopes. The relationship between the accuracy of sensors and the correlation factor is given by Bayard [34]. According to the literature [34], if the correlation does not exist between the gyroscopes, the drift is $1/\sqrt{3}$ of that of a single gyroscope. In general, the separate MEMS gyroscopes are independent of each other, and their measurements do not affect each other. Therefore, it can be assumed that there is no correlation between gyroscopes because the sensors are identical in design and operating conditions [29].

The gyroscope systematic error caused by bias, scale factors and misalignments can be compensated via an on-board Kalman filtering approach [36]. However, gyroscope random noise sources such as quantization noise (QN), angular random walk (ARW), rate random walk (RRW), and bias instability (BI) are not easily estimated by the same way, due to their random characteristic. In the flight of high-spin projectiles, the random error model of MEMS gyroscopes contains not only ARW and RRW, but also other noise items. Moreover, the MEMS gyroscope’s output signal has a weak linear trend item [25]. Furthermore, the output random signal is a non-stationary time sequence [26]. Therefore, the improved gyroscope random error model based on the ARIMA model is proposed, as shown in Equation (24).

(24) $$\left\{ \begin{array}{l} \dot{B}\left(t\right)=N_{r}\left(t\right)\\ Y\left(t\right)=\omega \left(t\right)+B\left(t\right)+x_{k}+N_{a}\left(t\right)\\ x_{k}=1.2683x_{k-1}-0.5099x_{k-2}+0.2416x_{k-3}+a_{k}+0.6754a_{k-1} \end{array}\right.,$$

where $Y\left(t\right)={\left[y_{1},y_{2},y_{3}\right]}^{T}$, $B\left(t\right)={\left[b_{1},b_{2},b_{3}\right]}^{T}$, $N_{a}\left(t\right)={\left[n_{a1},n_{a2},n_{a3}\right]}^{T}$, $N_{r}\left(t\right)={\left[n_{r1},n_{r2},n_{r3}\right]}^{T}$. Y(t) composed of three gyroscope measurements is a three dimensional vector. B(t) is the drift of gyroscopes placed on the $X_{b}$-axis. $N_{a}\left(t\right)$ and $N_{r}\left(t\right)$ are ARW and RRW noise vectors, respectively.

### 3.3. Sage-Husa Adaptive Kalman Filter Design

In the low dynamic state, the CKF is used to fuse MEMS gyroscope output data. The premise of CKF to obtain the optimal estimation is that the structural parameters and statistical noise parameters of stochastic dynamic systems need to be known accurately. However, in the flight condition of the high dynamic of the projectile, the process noise of MEMS gyroscope is often time-varying or unknown. Therefore, the adaptive filtering method with process noise time-varying estimator is introduced into of MEMS gyroscope data fusion algorithm [37]. According to the modified random error model, a Sage-Husa Adaptive Kalman Filter (SHAKF) that can suppress the time-varying process and measurement noise under the flight condition of the high dynamic of the projectile is designed for the fusion of MEMS gyroscope output data. The state vectors is shown in Equation (25) [38].

(25) $$X={\left[b_{1},b_{2},b_{3},x_{k},x_{k-1},x_{k-2},\omega \right]}^{T},$$

ω is real angular rate. and is modeled as white noise driven by $n_{\omega }$.

(26) $$\dot{\omega }=n_{\omega },$$

(27) $$E\left[n_{\omega }\left(t\right)n^{T}{}_{\omega }\left(t+\tau \right)\right]=q_{\omega }\delta \left(\tau \right),$$

where $n_{\omega }$ is a delta-correlated noise process, $q_{\omega }$ is the variance of $n_{\omega }$. According to Equation (24), the state equation is described by the following equation:

(28) $$\dot{X}\left(t\right)=F\left(t\right)\cdot X\left(t\right)+G\left(t\right)\cdot W\left(t\right),$$

where

(29) $$W\left(t\right)={\left[n_{r1},n_{r2},n_{r3},a_{k},a_{k-1},n_{\omega }\right]}^{T},$$

The discrete-time form of the system process model can be described as:

(30) $$X_{k}=\Phi_{k/k-1}X_{k-1}+\Gamma_{k/k-1}W_{k-1},$$

where $\Phi_{k/k-1}$ is the state transition matrix, $\Gamma_{k/k-1}$ is the system noise distribution matrix and $W_{k-1}$ is system noise vector. In the Equation (30), the dimension of $\Phi_{k/k-1}$ is 7×7, and $\Phi_{k/k-1}$ is exactly as shown in Equation (31). The dimension of $\Gamma_{k/k-1}$ is 7×6, and $\Gamma_{k/k-1}$ is shown in Equation (32).

(31) $$\Phi_{k/k-1}=\left[\begin{array}{ccccccc} 1 & 0 & 0 & 0 & 0 & 0 & 0\\ 0 & 1 & 0 & 0 & 0 & 0 & 0\\ 0 & 0 & 1 & 0 & 0 & 0 & 0\\ 0 & 0 & 0 & 1+\varphi_{1} & \varphi_{2}-\varphi_{1} & -\varphi_{2} & 0\\ 0 & 0 & 0 & 1 & 0 & 0 & 0\\ 0 & 0 & 0 & 0 & 1 & 0 & 0\\ 0 & 0 & 0 & 0 & 0 & 0 & 0 \end{array}\right],$$

(32) $$\Gamma_{k/k-1}=\left[\begin{array}{cccccc} T_{s} & 0 & 0 & 0 & 0 & 0\\ 0 & T_{s} & 0 & 0 & 0 & 0\\ 0 & 0 & T_{s} & 0 & 0 & 0\\ 0 & 0 & 0 & 1 & -\theta_{1} & 0\\ 0 & 0 & 0 & 0 & 0 & T_{s}\\ 0 & 0 & 0 & 0 & 0 & 0\\ 0 & 0 & 0 & 0 & 0 & 0 \end{array}\right],$$

$T_{s}$ represents the system sample interval.

The output of MEMS gyroscope is selected as the measurement, which are as shown in Equation (33).

(33) $$Z_{k}={\left[y_{1},y_{2},y_{3}\right]}^{T},$$

The observation equation has the following form:

(34) $$Z_{k}=H_{k}X_{k}+V_{k},$$

where $H_{k}$ is the transformation matrix linking the state vector and the measurement vector, and $V_{k}$ denotes the measurement noise vector.

(35) $$H_{k}=\left[\begin{array}{ccccccc} 1 & 0 & 0 & 1 & 0 & 0 & 1\\ 0 & 1 & 0 & 1 & 0 & 0 & 1\\ 0 & 0 & 1 & 1 & 0 & 0 & 1 \end{array}\right],$$

(36) $$V_{k}={\left[n_{a1},n_{a2},n_{a3}\right]}^{T},$$

In the Equations (30) and (34), $W_{k-1}$ and $V_{k}$ both are Gaussian white noise: $W_{k-1}∼WN\left(q_{k},Q_{k}\right)$, $V_{k-1}∼WN\left(r_{k},R_{k}\right)$, which are independent of each other and with time varying mean and covariance matrix time-varying mean and covariance matrix. They meet the following requirements [39]:

(37) $$\begin{array}{c} E\left[W_{k}\right]=q_{k},E\left[W_{k}W_{j}^{T}\right]=Q_{k}\delta_{kj}\\ E\left[V_{k}\right]=r_{k},E\left[V_{k}V_{j}^{T}\right]=R_{k}\delta_{kj}\\ E\left[W_{k}V_{j}^{T}\right]=0 \end{array},$$

**Figure 3:**
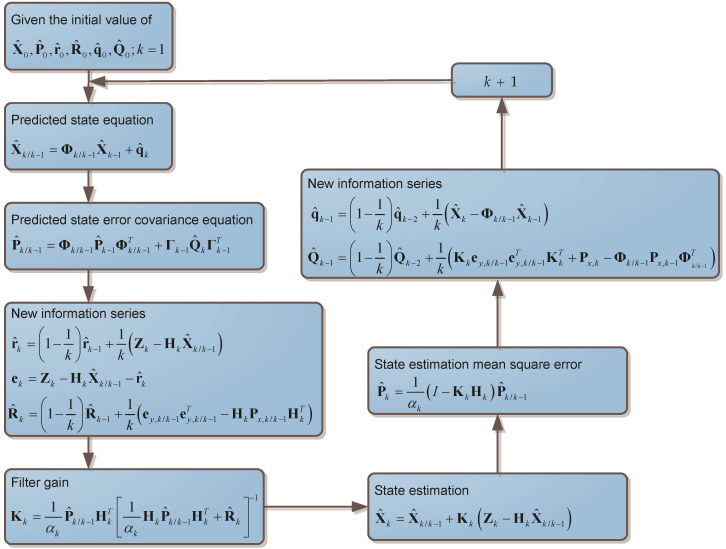
Flow chart of the Sage–Husa Adaptive Kalman Filter (SHAKF) algorithm.

The flow chart of the SHAKF algorithm is shown in Figure 3. 

Where $\alpha_{k}\in (0,1]$ is the adaptive scale factor and $K_{k}$ is the SHAKF gain. The adaptive factor $\alpha_{k}$ is helpful to balance the observations and predicted states. In the SHAKF algorithm, an adaptive factor constructed by three-segment function of discrepancy is constructed by [40]

(38) $$\alpha_{k}=\{\begin{array}{cc} 1 & \left|\Delta {\tilde{X}}_{k}\right|\leq c_{0}\\ \frac{c_{0}}{\left|\Delta {\tilde{X}}_{k}\right|}{\left(\frac{c_{1}-\left|\Delta {\tilde{X}}_{k}\right|}{c_{1}-c_{0}}\right)}^{2} & c_{0}<\left|\Delta {\tilde{X}}_{k}\right|\leq c_{1}\\ 0 & \left|\Delta {\tilde{X}}_{k}\right|>c_{1} \end{array},$$

where $1\leq c_{0}\leq 1.5$ and $3\leq c_{1}\leq 4.5$ are two constants. The learning statistics of the predicted state error model $\Delta {\tilde{X}}_{k}$ is expressed as

(39) $$\left|\Delta {\tilde{X}}_{k}\right|=\frac{\left\|{\hat{X}}_{k}-{\hat{X}}_{k/k-1}\right\|}{\sqrt{tr\left({\hat{P}}_{k/k-1}\right)}},$$

where tr(•) represents the trace of the matrix.

By defining the selection vector $e_{7}$, the real angular rate signal can be obtained as follows:

(40) $$w=e_{7}^{T}\cdot X,$$

where $e_{7}={\left[\begin{array}{cc} 0_{1\times 6} & 1 \end{array}\right]}^{T}$. 

## 4. Simulation and Experiment Results

In this section, simulation and experiment are used to verify and evaluate the performance of the proposed OBARS method. In this method, three ADXRS649 MEMS gyroscopes are placed parallel to the $X_{b}$-axis to improve the measurement accuracy of the roll axis. Firstly, the experiment is implemented to verify and evaluate the performance of the OBARS method in static state. Then, the superiority of the OBARS method in the high-spin state is discussed by the trajectory simulation and turntable experiment. 

### 4.1. Performance Assessment in Static

The raw data of gyroscope was collected at a sampling frequency of 100 Hz for 2 hours under the stationary. The test was held at room temperature. In the static experiment, the proposed OBARS method is compared with the traditional arithmetic mean method and CKF. In the traditional arithmetic average method, the mean value of three gyroscope output signals is taken as the optimal angular rate. In the CKF algorithm, the conventional random error model of gyroscope usually is selected as the state space model. The SHAKF algorithm is applied to the fusion of MEMS gyroscopes data of roll axis. In the SHAKF algorithm, initial value of state ${\hat{X}}_{0}$ and its variance matrix ${\hat{P}}_{0}$ are selected as zero and unity, respectively. In real-time, the measurement noise $R_{k}$ and covariance matrix of system noise $Q_{k}$ vary with time. The adaptive factor $\alpha_{k}$ is adapted at each time step of iteration to estimate the new value in the signal. 

The Allan variance (AV) is adopted to identify and extract random error terms [41]. Furthermore, the results denoted by $\sigma_{A}^{2}\left(\tau \right)$ of AV analysis for gyroscope error can be written as

(41) $$\begin{array}{cc} \sigma_{A}^{2}\left(\tau \right) & =\sigma_{BI}^{2}\left(\tau \right)+\sigma_{ARW}^{2}\left(\tau \right)+\sigma_{RRW}^{2}\left(\tau \right)+\sigma_{QN}^{2}\left(\tau \right)+\sigma_{RR}^{2}\left(\tau \right)\\ & =\frac{4B^{2}}{9}+\frac{N^{2}}{\tau }+\frac{K^{2}\tau }{3}+\frac{3Q^{2}}{\tau^{2}}+\frac{R^{2}\tau^{2}}{2} \end{array}$$

where B,N,K,Q,R represent the coefficient of BI, ARW, RRW, QN and RR, respectively. τ is the correlation time. The characteristics of each error item are summarized in Table 1.

**Figure 4:**
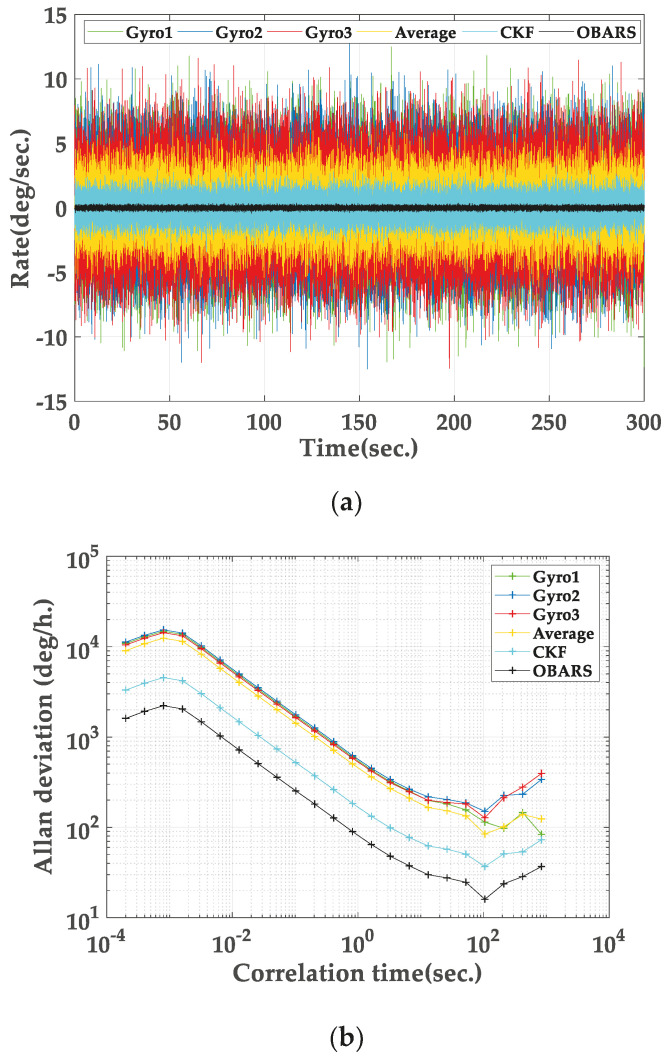
(**a**) Micro-Electro-Mechanical System (MEMS) gyroscope signal and fusion results; (**b**) Corresponding Allan variance plot.

MEMS gyroscope signal and fusion results are shown in Figure 4. The Allan variance analysis for the whole data set and all the methods is plotted in Figure 4b. From the AV analysis, the performance of all methods is compared.

It can be clearly seen that the accuracy of angular rate measurement can be improved by fusing the output signal of gyroscopes. From the Allan variance plot, the noise terms of the angle random walk (ARW) and the bias instability (BI) are the dominated noise sources. According to Table 1, the ARW noise has a slope of -1/2. Similarly, the BI noise is indicated as the zero slope in the log-log AV plot. From Figure 4b, the noise terms of the ARW and BI are extracted from the −1/2 and zero slopes respectively. The ordinate $\sigma_{ARW}$ of the intersection point of a log–log plot of the Allan standard deviation (or extension line) with slope -1/2 and the straight line τ=1 is ARW. The ordinate $\sigma_{BI}$ of the intersection point of a log–log plot of the Allan standard deviation (or extension line) with slope 0 and the straight line τ=1 is $\sqrt{2\ln2/\pi }B\approx 0.664$. In general, the bottom of the plot is generally selected as the point with a slope of 0. In the Table 2, the performance of the all the methods is also evaluated using the AV for the MEMS gyroscope signal before and after fusing. 

Variance $\sigma^{2}$ is used to describe the degree of dispersion of the data set from the true angular rate as follows:

(42) $$\sigma^{2}=\frac{\sum {\left(x-\mu \right)}^{2}}{N},$$

where x represents the whole data set, μ represents the real angular rate in the stationary, i.e., μ=0, and N is the number of data points.

According to the results, it can be clearly seen that the ARW and BI noise are reduced approximately by an order of 1 when using the OBARS method. Therefore, the OBARS method of combining the outputs of multiple gyroscopes can give much more accurate results than those for a single gyroscope. 

### 4.2. Performance Assessment in Dynamic

#### 4.2.1. Performance Analysis Using Simulation Data

**Figure 5:**
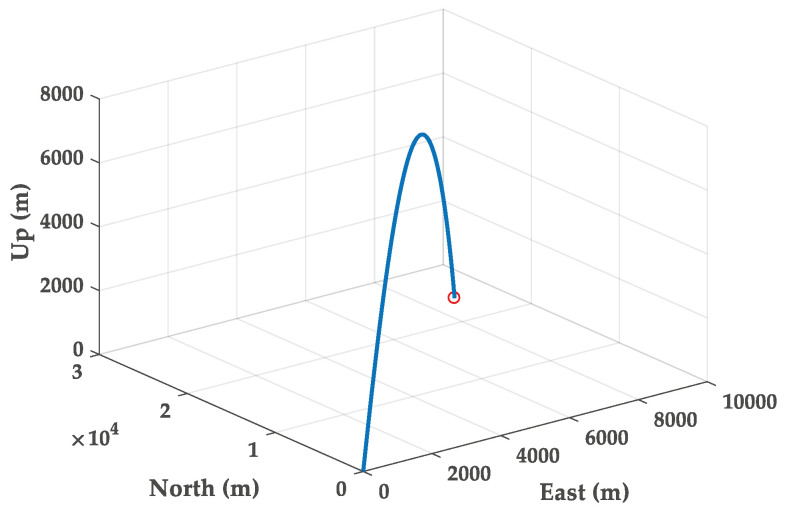
Real trajectory of the projectile.

**Figure 6:**
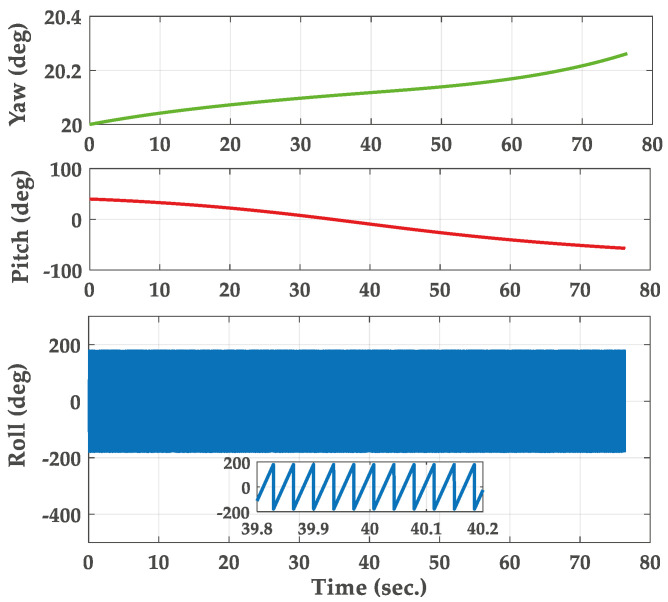
Real attitude angle of the projectile.

**Figure 7:**
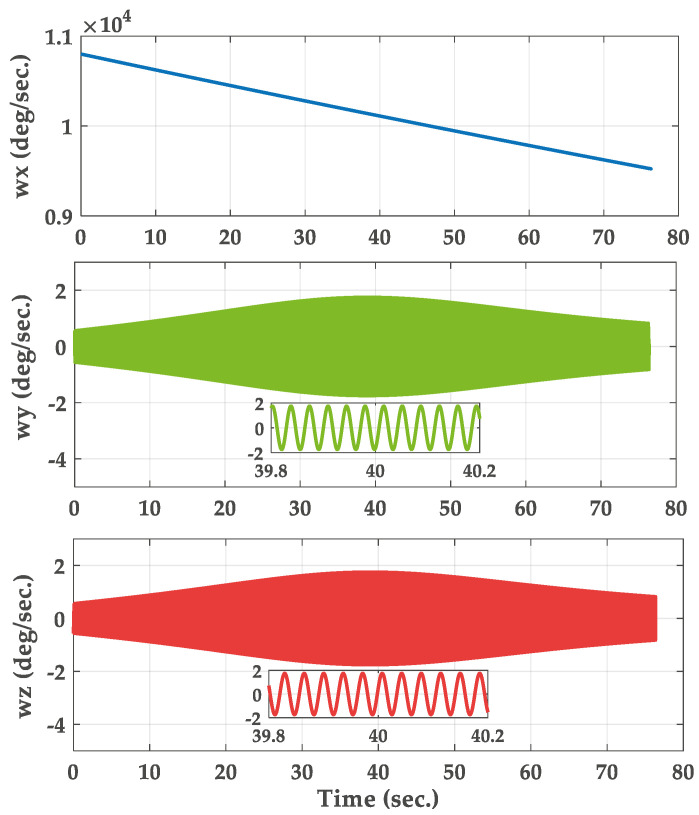
Real angular rate of the projectile.

In this section, the trajectory generation program is used to generate the theoretical trajectory of projectile. The feasibility of roll rate measurement system for high-spin projectile is verified by simulation. The simulation parameters are as follows. The mass and length of the projectile are 45 kg and 1.5 m, respectively. The polar moment of inertia of the projectile is $0.8\;\mathrm{kg}*m^{2}$. The pressure is $10^{5}\;\mathrm{kPa}$. The initial velocity of the projectile is set to 800 s/m. The initial yaw, pitch and roll angles are $20^{\circ }$, $40^{\circ }$ and $0^{\circ }$. The longitude and latitude of the launch site are $112.5^{\circ }\;E$ and $38.1^{\circ }\;N$. The muzzle angular rate is set to $10,800{}^{\circ }/s$. The sampling frequency is chosen to be 5 kHz and the total simulation time is 80 s. The three-dimensional trajectory of the projectile is shown in Figure 5. The projectile flight reaches 7168 m in altitude. Figure 6 gives the real attitude angle of the projectile. The roll angle presents a dense periodic variation in the flight process. The real angular rate of the projectile in ballistic flight is provided in Figure 7. 

**Figure 8:**
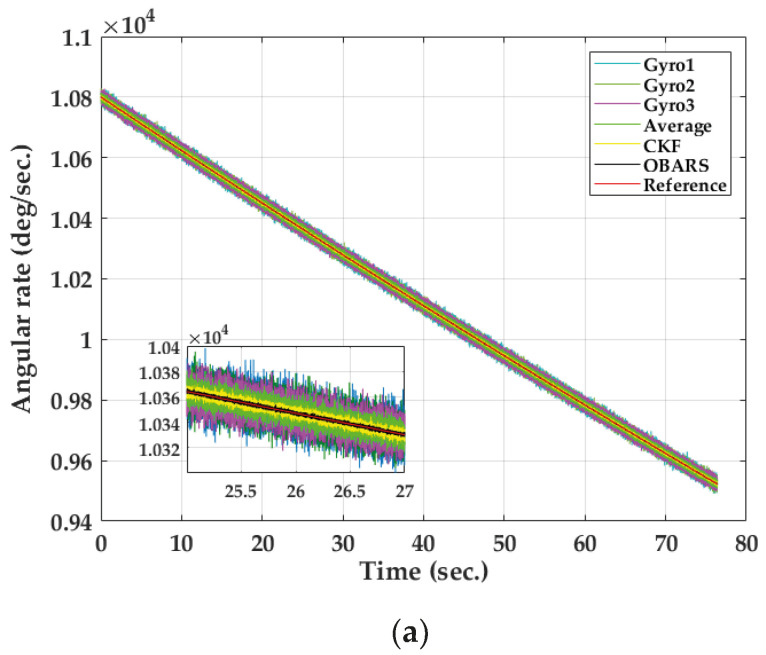

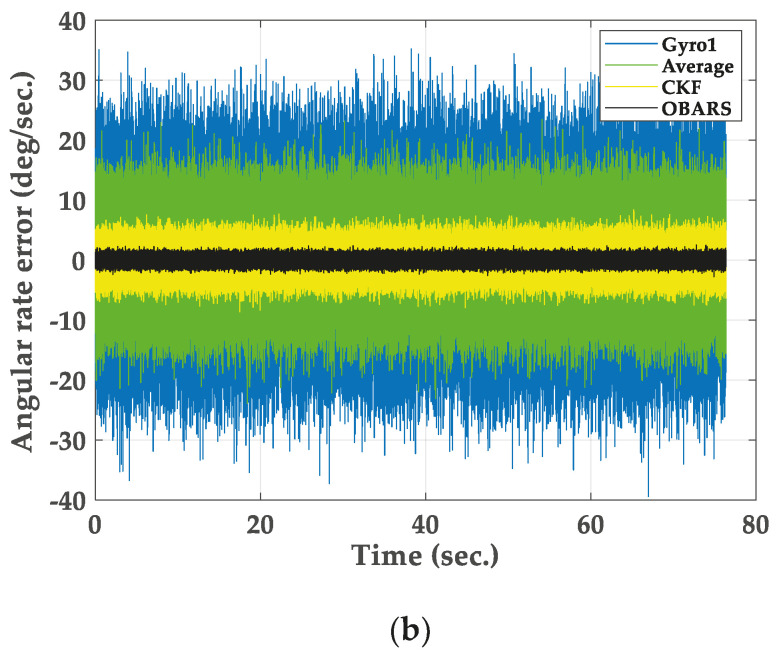
(**a**) Simulation results of angular rate; (**b**) Simulation results of angular rate error.

The white noise of $254{}^{\circ }/h$, $257{}^{\circ }/h$ and $261{}^{\circ }/h$ (ARW) is seeded in Gyro1, Gyro2 and Gyro3, respectively. The bias of Gyro1, Gyro2, and Gyro3 is set to $137{}^{\circ }/h$, $138{}^{\circ }/h$ and $140{}^{\circ }/h$. Filtering results are observed before and after fusing are shown in Figure 8a. The error between the measured value and the real value is provided in Figure 8b. In the simulation, the angular rates measured by the three gyroscopes are almost the same. For ease of observation, only the angular rate error of Gyro1 is presented.

Figure 8a shows the superiority of the proposed method in the process of simulating projectile flight. It can be seen that the overall flight time of the projectile is about 80 s, and the maximum rolling angular rate of the gyroscope measured is $10,800{}^{\circ }/s$. The blue, light-green, and purple lines represent the output of Gryo1, Gryo2, and Gryo3 after seeding noise, respectively. The green, yellow and black lines represent the angular rate after fusing, and the red lines represent the ideal angular rates of gyroscope. According to the results, it can be clearly seen that the angular rate error of Average and CKF with the traditional method is very large. The performance of both methods is obviously improved by the OBARS method. Simulation results show that the estimation accuracy of roll angular rate of the projectile is significantly improved by using the OBARS method. It also can be seen that CKF has better performance than Average. 

**Figure 9:**
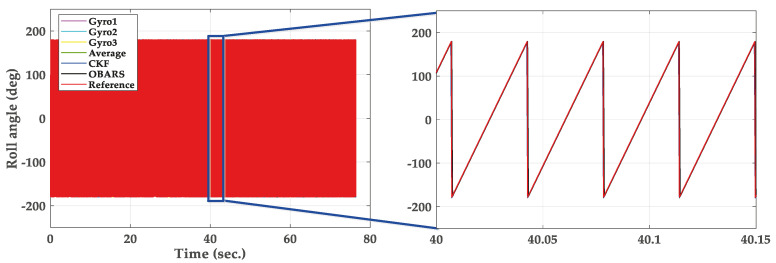
Simulation results of roll angle. The right figure is a local enlarged drawing.

**Figure 10:**
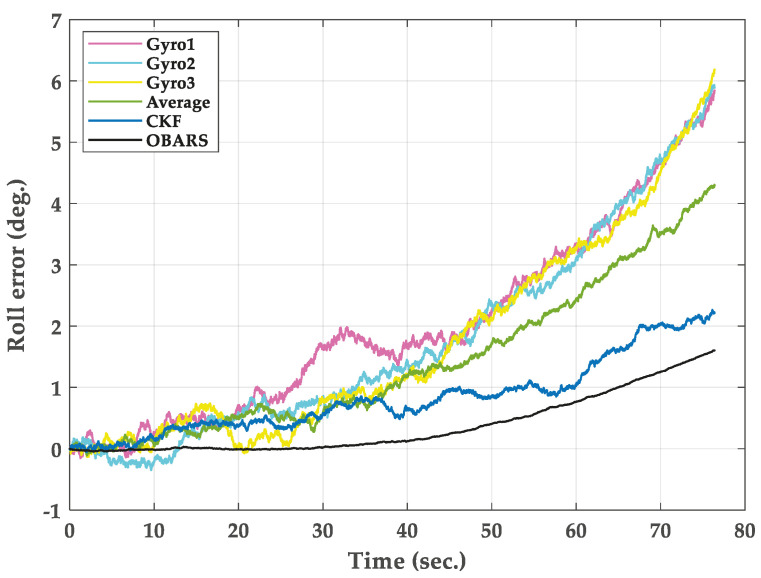
Simulation results of roll error.

Figure 9 shows the roll angle solved by before and after fusion. The right is a local enlarged drawing of the roll angle. Figure 10 shows the solved error of roll angle. The maximum roll angle error solved by Gyro1, Gyro2, and Gyro3 is $6{}^{\circ }/s$, and the error showed a trend of divergence. The error is about $1.8{}^{\circ }/s$ when the OBARS method is applied to gyroscopes signal. It can be directly seen from Figure 10 that the roll angular rate error changes changes smoothly after using the OBARS method.

#### 4.2.2. Performance Analysis Using Experimental Data

**Figure 11:**
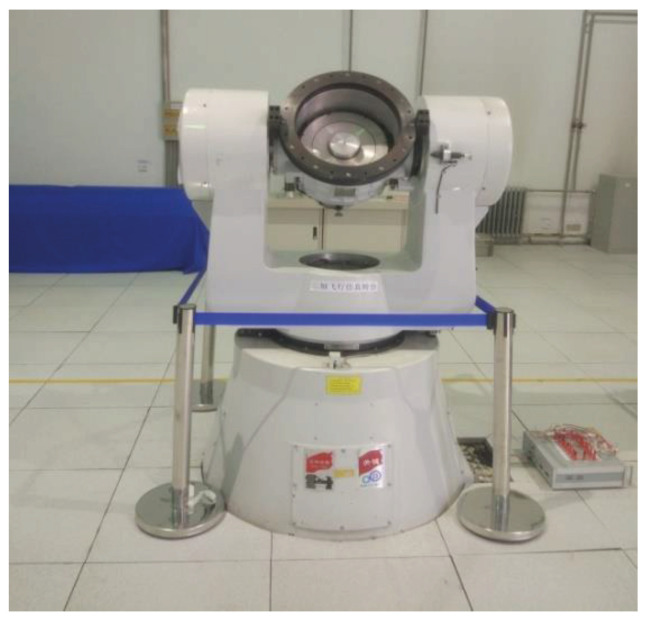
The experimental equipment.

In this section, the turntable experiment is implemented as shown in Figure 11. The IMU with the optimal gyroscope geometry layout is mounted on high-speed flying simulation turntable used to simulate the separation state of high-spin projectile. The calibration ensures that the roll axis of the system is coaxial with that of the turntable, and that there is no shaking during the experiment. The turntable is controlled according to a given control command. The turntable parameters used in the experiment are shown in Table 3. Accordingly, the output angular rates of three single gyroscopes are recorded. The data was obtained at a sampling frequency of 5 kHz. 

**Figure 12:**
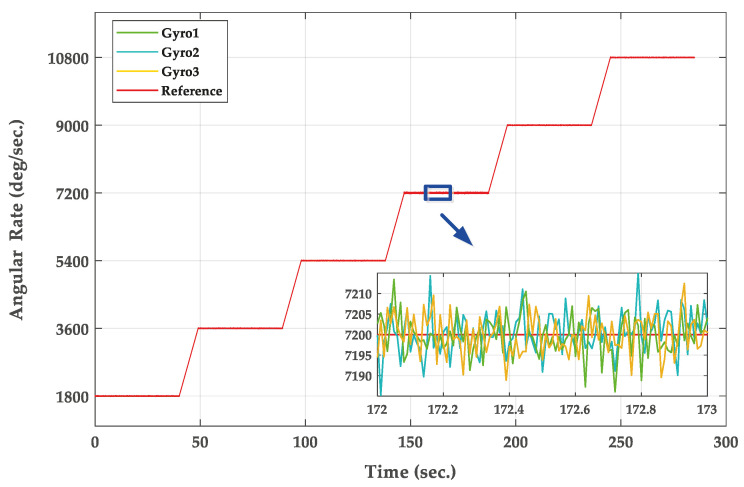
The output of MEMS gyroscopes.

The flight attitude of the projectile is simulated by controlling the attitude change of the turntable. In order to verify the accuracy of the actual angular rate, the sensors output data are collected with the turret. The inner frame of the three-axis flight turntable simulates the rolling motion of the projectile at an rotational speed of 5 r/s, 10 r/s, 15 r/s, 20 r/s, 25 r/s and 30 r/s. The actual output of the three single gyroscopes is shown in Figure 12.

The turntable only runs the roll axis, the pitch axis and the yaw axis are not given control commands. Therefore, the theoretical outputs of the Y-axis and Z-axis gyroscope are zero. The output of the roll axis gyroscope is shown in Figure 12, compared with the real value, the measuring error of gyroscope is $10{}^{\circ }/s$. For the traditional single gyroscope measurement method, the bias error of inertial sensors will be accumulated over time and resulting in huge calculation errors of angular rate measurement. Therefore, the traditional single gyroscope measurement method is not able to estimate the angular rate accurately when the bias cannot be ignored.

**Figure 13:**
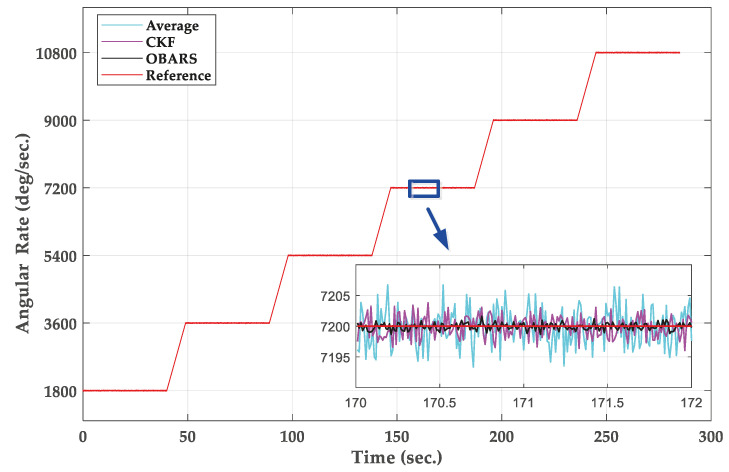
The angular rate output of based on Average, Conventional Kalman Filter (CKF), and optimization-based angular rate estimation (OBARS).

Figure 13 show the variation of the roll angular rate for three methods which are, respectively, Average, CKF and OBARS. All methods are applied to the fusion of three single-gyroscope signals. When the inner frame of turntable is operated at an rotational speed of 20r/s, the error of angular rate of the OBARS method is $\pm 1^{\circ }/s$ compared with the theoretical value. Furthermore, the OBARS method is closer to the real value compared with the Average and CKF. According to the result, it can be clearly seen that the dynamic error are stable in a certain interval and do not diverge with time.

The root mean squared error (RMSE) is used as the evaluation criteria to test the fusion performance in the dynamic condition [41]. The RMSE values are calculated for the SHAKF algorithm before and after fusion of MEMS gyroscope sensor signals. It is defined as

(43) $$RMSE=\sqrt{\frac{1}{N}{\sum_{i=1}^{N}\left(A_{i}-{\hat{A}}_{i}\right)}^{2}},$$

**Figure 14:**
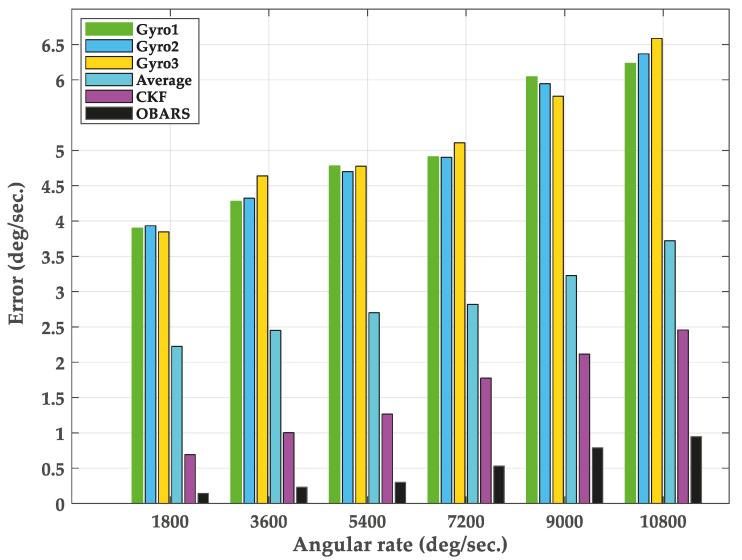
The root mean squared error (RMSE) results of different angular rate.

The RMSE values are evaluated for for all the methods and shown in Figure 14. 

In Figure 14, the cyan, purple, and black rectangular boxes represent the RMSE values of angular rate obtained by arithmetic mean, CKF and OBARS methods, respectively. The green, blue and yellow rectangular boxes represent the RMSE values of angular rate of three gyroscopes respectively. From these figures, the estimation error of single gyroscope is much higher than that of the three methods. It can be clearly seen that the angular rate error of Average and CKF with the traditional method is very large. Furthermre, the proposed OBARS method has better accuracy. The experimental system can verify the roll rate measurement algorithm presented in this paper.

## 5. Conclusions

High precision measurement of the attitude of the projectile is the key technology in traditional artillery guidance. Restricted by the special environment such as high rotation, the measurement of roll angular rate of the projectiles has always been a challenge. This paper proposed a optimization-based angular rate estimation (OBARS) method applied to high-spin projectiles. In the method, the SHAKF algorithm based on the hybridization of the gyroscope error model and ARIMA model can suppress the time-varying process and measurement noise under the flight condition of the high dynamic of the projectile. Therefore, the accuracy of angular rate measurement can be improved. In order to verify the validity and accuracy of the method, simulation and experiments are carried out in both static and dynamic. From the simulations and experiments above, the proposed method is superior to the traditional angular rate measurement method. This article draws the following conclusions:

1. In order to update the attitude of projectile by using angular rate information measured by gyroscope in real time, the accuracy of sensor should be high enough, and the higher it is, the smaller the error of attitude angle of projectile is.
2. The optimal geometric layout of gyroscopes of the inertial measurement unit (IMU) suitable for the high dynamic of high-spin projectiles is designed. In this configuration method, the output angular rate model of redundant gyroscope system based on ARIMA is established, and then the conventional random error model is improved with the ARIMA model. The maximum amount of accuracy can be extracted from a given number of redundant single-degree-of-freedom gyroscopes with optimal geometric configurations.
3. The SHAKF algorithm is designed to suppress the time-varying process and measurement noise under the flight condition of the high dynamic of the projectile. It can reduce noise and minimize bias instability. In addition, the robustness of the system is also enhanced.

## Figures and Tables

**Table 1:** Noise characteristics of gyroscope.

Noise Type	Allan Variance	Units	Slope
Quantization noise	$\sigma_{QN}^{2}\left(\tau \right)=3Q^{2}/\tau^{2}$	(∘/s)	1
Angle rate random walk	$\sigma_{ARW}^{2}\left(\tau \right)=N^{2}/\tau$	$\left(\circ /\sqrt{h}\right)$	−1/2
Bias instability	$\sigma_{BI}^{2}\left(\tau \right)=2\ln2B^{2}/\pi$	(∘/h)	0
Rate random walk	$\sigma_{RRW}^{2}\left(\tau \right)=K^{2}\tau /3$	$\left(\circ /h/\sqrt{h}\right)$	1/2
Rate ramp	$\sigma_{RR}^{2}\left(\tau \right)=R^{2}\tau^{2}/2$	$\left(\circ /h^{2}\right)$	1

**Table 2:** Allan variance analysis results.

Method	BI $\left({}^{\circ }/h\right)$	ARW $\left({}^{\circ }/\sqrt{h}\right)$	Variance ${\left(\circ /s\right)}^{2}$
Gyro1 raw data	$1.37\times 10^{2}$	$4.31\times 10^{2}$	34.0068
Gyro2 raw data	$2.18\times 10^{2}$	$4.52\times 10^{2}$	37.1571
Gyro3 raw data	$1.85\times 10^{2}$	$4.18\times 10^{2}$	31.8644
Average	$1.21\times 10^{2}$	$3.52\times 10^{2}$	23.9088
CKF	55.47	$1.31\times 10^{2}$	3.2253
OBARS	19.92	62	0.7649

**Table 3:** Performance Parameters of Turntable.

Angular Rate Resolution	Angular Rate Accuracy	$\mathrm{Angular}\;\mathrm{Rate}\;\mathrm{Range}\;\left({}^{\circ }/s\right)$		
		Roll	Pitch	Yaw
0.001%	0.01%	0.01∼12,000	0.01∼400	0.01∼400
